# Effect of the Brief Instructional Video Intervention on the Quality of Cardiopulmonary Resuscitation

**DOI:** 10.7150/ijms.79433

**Published:** 2023-01-01

**Authors:** Fang Li, Cheng-Pang Yang, Chun-Hao Chang, Chia-An Ho, Cheng-You Wu, Hung-Chih Yeh, Chun-Wei Hsu, Pei-Jung Chang, Chin-Shan Ho

**Affiliations:** 1 School of Physical Education, Central China Normal University, Wuhan, China.; 2 Department of Orthopedic Surgery, Division of Sports Medicine Chang Gung Memorial Hospital, College of Medicine, Chang Gung University, Linkou, Taiwan.; 3 Graduate Institute of Sports Science, National Taiwan Sport University, Taoyuan, Taiwan.; 4 College of Exercise and Health Science, National Taiwan Sport University, Taoyuan City, Taiwan.; 5 Department of Anesthesiology, Sin-Lau Hospital, Taiwan.

**Keywords:** cardiopulmonary resuscitation, chest compression, out-of-hospital cardiac arrest, fatigue

## Abstract

**Background:** Chest compressions are the basis of cardiopulmonary resuscitation (CPR), and high-quality chest compressions can improve survival rate in patients with out-of-hospital cardiac arrest. Although many efforts have been made to improve the quality of CPR in inexperienced adults, the results are still not high, especially during emergencies. The primary purpose of this study is to investigate whether a brief instructional chest compression-only CPR video could improve chest compression quality in inexperienced adults.

**Methods:** One hundred adults with no CPR experience (age: 20.28 ± 2.28 years; women: 50, men: 50) participated in this study. Participants completed body composition and handgrip strength measurements, and performed two CPR quality tests on the Laerdal^®^ Little Anne QCPR Manikin, namely without video-CPR (WV-CPR) and video-CPR (V-CPR). The WV-CPR quality test was performed first. After 2 minutes of continuous chest compression, the participants rested for 10 seconds and repeated 3 cycles (phase 1, phase 2, and phase 3). After resting for more than 72 hours, V-CPR quality test was conducted. During the V-CPR with video intervention, the participants also continued to compress the chest for 2 minutes, and then rested for 10 seconds, repeating 3 cycles.

**Results:** In phase 1, compared with WV-CPR, the V-CPR has a significant increase (*p* < 0.001) in chest compression fraction (CCF) (56.31 ± 33.22% vs. 41.82 ± 32.30%) and percent of correct compression rate (PCCR) (96.17 ± 8.45% vs. 26.31 ± 37.55%). In addition, the V-CPR has significantly lower (*p* < 0.001) chest compression rate (CCR) (110.85 ± 2.40 cpm vs. 128.86 ± 24.52 cpm) and rating of perceived exertion (RPE) (11.89 ± 2.25 vs. 12.87 ± 2.25). For phases 2 through 3, V-CPR and WV-CPR achieved significant differences in CCF, CCD, CCR, PCCR, and RPE (*p* < 0.01). There were significant differences (*p* < 0.05) in CCF, CCD, chest compression rebound rate, and RPE among the different administration stages of both WV-CPR and V-CPR.

**Conclusions:** The results of this study revealed that a brief instructional chest compression-only CPR video could improve chest compression quality for inexperienced adults by reducing fatigue and CCR, and increasing CCF and PCCR.

## Introduction

Sudden cardiac arrests are the most common cause of death in the world 1, and most of these events occur in out-of-hospital such as the home or some private setting 2-4. Despite current advances in medical technology, the survival rate of out-of-hospital cardiac arrest (OHCA) patients remains below 10% 5-7. Cardiopulmonary resuscitation (CPR) is one of the most important treatment options for patients with OHCA 1. Bystander high-quality CPR contributes to improved survival in patients with OHCA 8, 9, including adequate chest compression rate (CCR), chest compression depth (CCD), and full chest recoil, as well as interruption reduction in chest compressions and prevention of excessive ventilation 2. While chest compression is the most important and fundamental skill in effective CPR 10, compression-only CPR is strongly recommended by the American Heart Association (AHA) for inexperienced rescuers 7, 2. According to the 2020 AHA's recommended guidelines, rescuers should perform chest compression for adults in cardiac arrest at a rate of 100-120 compressions per minute (cpm), and CCD should be maintained at 5-6 cm. Greater compression depths can result in rib fractures, sternum fractures, and heart or lung damage 11. Complete chest recoil is also important during CPR, which improves hemodynamics by generating relatively negative intrathoracic pressure, thereby pumping venous blood back to the heart, providing cardiac preload before the next chest compression phase 12, 13. In addition, in order to obtain effective systemic blood flow and coronary perfusion through chest compressions, the frequency and duration of interruptions in chest compressions should also be minimized 7, 14-16.

In the out-of-hospital setting, neither emergency ambulance technicians nor laypeople can always perform CPR according to AHA guidelines 17-19. The chest compressions they provide often suffer from insufficient depth, excessive interruptions, excessive ventilations, and compression rates that are too fast. Even with the hand positions recommended by the AHA guidelines and instructing the rescuer to allow full chest recoil, it is difficult to achieve full chest recoil in CPR practice 13. Past studies have demonstrated that CCD and complete chest recoils were negatively correlated with CCR 12, 20, and compression rates above 120 cpm are associated with adverse effects for compression depth and complete chest recoils 21-23. While height, weight, body mass index (BMI), and muscle strength are positive predictors of compression depth 24-26, in general men's compression depth is greater than that of women's 17, 18, 26, 27. The 2020 AHA guidelines recommend alternating rescuers every 2 minutes or every 200 consecutive chest compressions during CPR to maximize the percentage of adequate compressions 8, 14. In the single-rescuer scenario, although emergency medical services (EMS) response times vary widely, they generally exceed 2 minutes, so rescuers may need to continuously perform chest compressions for 5 to 10 minutes or more 8. Prolonged continuous compressions lead to increased fatigue 28, 29, which prevents rescuers from maintaining adequate depth and rate of compressions throughout the EMS reaction time, CCF and chest compression rebound rate (CCRR) also decreased with resuscitation time 8, 10, 20.

To improve the quality of CPR for untrained lay rescuers, the AHA emphasizes the need for emergency dispatchers to provide chest compression-only CPR guidance for callers of OHCA patients 30. In dispatch-assisted CPR (DA-CPR), the ambulance dispatcher keeps in touch with the rescuer continuously by telephone and describes the CPR compression hints to the rescuer in the form of voice, so that the rescuer can perform chest compressions step by step. DA-CPR instructions can greatly increase the likelihood of bystanders performing CPR and improve survival 2, 9, and are therefore becoming standard of care for witnessing OHCA 8. But when DA-CPR orders are initiated, only a few callers can complete the order and perform chest compressions before the arrival of EMS, mainly because of the longer time required for the diagnosis and guidance of cardiac arrests 9. Nowadays, with the development of 3C (computer, communication, and consumer electronics) products, many CPR feedback devices have been developed to improve the quality of CPR. Since rapid compression can easily lead to fatigue, which eventually leads to compression depth decrease, inaccurate hand position, lower correct compression rate and more severe incomplete chest compression 20, 27. Therefore, more attention should be paid to compression rate control during CPR. A metronome is an inexpensive and simple tool that can be used to guide compression rates 19. When using devices such as a metronome or song rhythm, the compression rate can be corrected to within the range recommended by guidelines 18, 19, 31. However, monitoring of compression depth is difficult. During chest compressions, a prompt or feedback device to measure sternal displacement is required to assist the rescuer in reaching the recommended depth of compression 32, but this device has not been widely used in clinical practice 18.

As a multimedia intervention, video has dual effects of vision and hearing. Interventional video during CPR is more able to attract the attention of lay bystanders and improve their learning efficiency, as well as their responsiveness to OHCA and chest compression skills 33. However, in the previous studies, video was rarely used to assist CPR compression quality 34, 35. Recent studies have demonstrated that the use of visual feedback devices during CPR can improve the percentage of correct compression depth, CCRR, correct hand position, and total CPR score, but not in the compression rate 36. Therefore, in this study, we designed a brief instructional CPR video, which covers reminders, explanations and demonstrations of 30-second CPR performance points, and provides a cadence (110 cpm) recommended by the 2020 AHA guidelines for later chest compressions to help chest compression providers more intuitively and effectively understand the hints of chest compression-only CPR action, and avoid the problem that the compression rate becomes faster or slower with time. The primary objective of this study is to investigate the effect of a brief instructional chest compression-only CPR video on compression quality of experienced adults in a single-rescuer scenario.

## Materials & Methods

### Study design

This study recruited adults without CPR experience to examine the effect of a brief video intervention on CPR quality in the single-rescuer scenario. According to statistics from the National Fire Agency of the Ministry of the Interior of Taiwan, the average response time of EMS is 6.2 minutes, which means that bystanders need to perform CPR compressions on OHCA patients for at least 6 minutes. Previous studies have shown that prolonged continuous compression can cause fatigue and affect compression quality, giving rescuers a 10-second rest period during continuous compression CPR can greatly improve chest compression quality 37. The 2020 AHA guideline recommendation was also taken into account that rescuers should be alternated every 2 minutes during CPR, and the interruption should not exceed 10 seconds 15. The scenario set in this study is the single-rescuer scenario. Therefore, we designed a 6-minute intermittent CPR quality test, that is, 3 phases (phase 1, phase 2, and phase 3) with a 10-second rest for every 2 minutes of continuous compression, and made a brief video according to the 2020 AHA revised CPR technical specifications (50-60 mm compression depth, 100-120 cpm compression rate, no interruptions, full chest recoil). In this study, the manikin used for CPR quality testing was Laerdal^®^ Little Anne QCPR (Laerdal Medical, Stavanger, Norway). The parameters on CCF, CCR, PCCR, CCD, and CCRR were calculated by the SimPad PLUS device (Laerdal, Stavanger, Norway) with activated SkillReporter software. This manikin study was approved by the Institutional Review Board of Fu Jen Catholic University (New Taipei City, Taiwan). Before the start of the CPR quality testing, we received informed consent from all participants.

### Participants

A total of 100 people without CPR experience participated in this study. All participants were randomly recruited via advertisements distributed in public spaces. Those with cardiovascular disease, as well as muscle and skeletal injuries identified by physicians as unsuitable for vigorous exercise were excluded. The body mass, percentage of body fat (PBF) and skeletal muscle mass of all participants were measured in this study using a body composition analyzer (InBody 570, Biospace, Inc. Seoul, Korea). The BMI is calculated by dividing body weight (kg) by the square of height (m). Handgrip strength was measured with a Takei 5401 handgrip digital dynamometer (Takei Scientific Instruments Co., Ltd, Tokyo, Japan).

### Intervention

**Figure 1:**
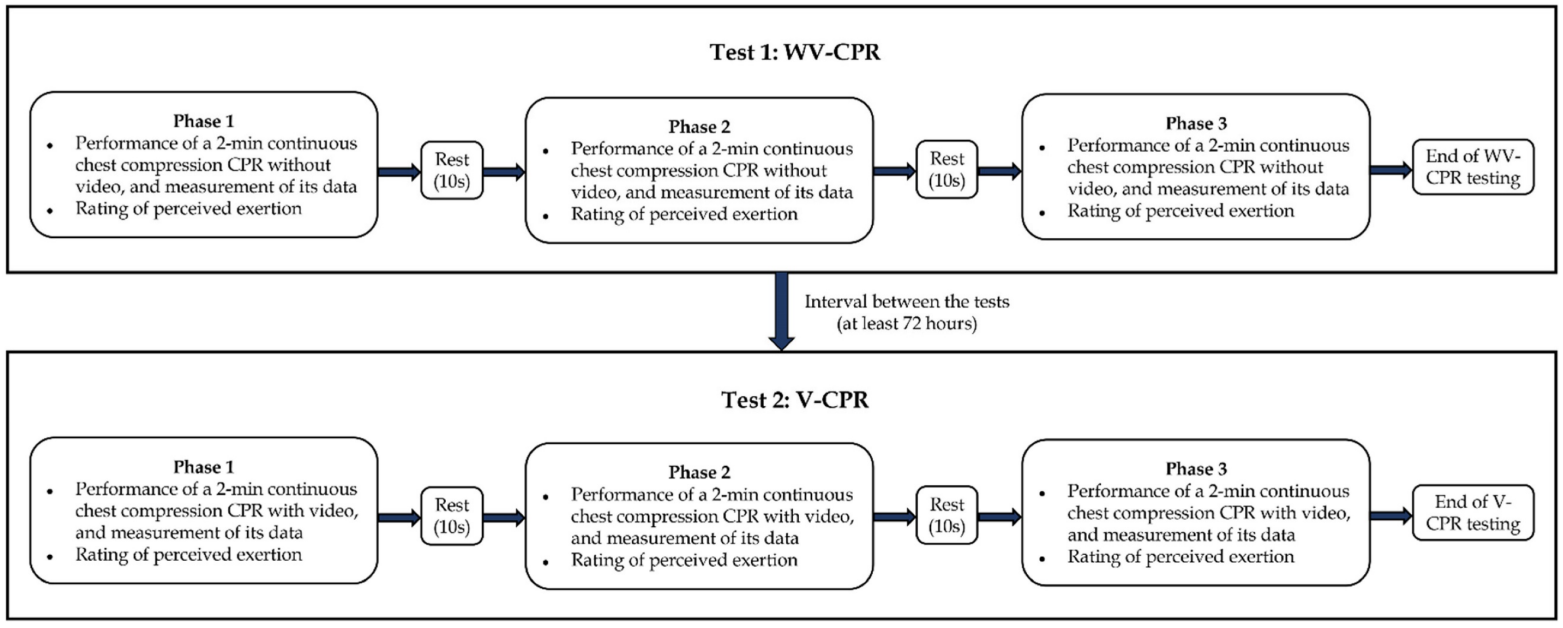
Flowchart of the experimental procedure.

In this study, each participant was required to perform two CPR quality tests on the Laerdal^®^ Little Anne QCPR Manikin, namely without video-CPR (WV-CPR) and video-CPR (V-CPR), with a rest for at least 72 hours between the two tests (Figure 1). Participants did not use any feedback device during the WV-CPR test. All participants performed chest compressions on the Laerdal^®^ Little Anne QCPR Manikin for 2 minutes (uninterrupted), followed by a 10-second rest, which was one set of chest compressions. The Borg rating of perceived exertion (RPE) scale (ratings 6-20) was used immediately after the execution stopped to ask participants how tired they are at the moment. A total of 3 sets of chest compressions were required to be completed in a row to complete the WV-CPR test.

During the V-CPR test, the sixth-generation IPAD (Apple Inc., California, United State of America) tablet was used to play chest compression-only CPR practice instructions for 30 seconds, and the compression rate (110 cpm) rhythm prompt tone video. The content of this video was designed in accordance with the 2020 AHA revised CPR technical specifications 15, and no other feedback devices were used in the whole process of V-CPR. Participants performed chest compressions on the Laerdal^®^ Little Anne QCPR Manikin for 2 minutes (uninterrupted) and then rested for 10 seconds to complete 1 set of chest compressions. The Borg RPE scale was used to ask the participant's current fatigue level immediately after the execution was stopped. A total of 3 sets of continuous chest compressions were required to be completed for the V-CPR test.

### Statistical Analysis

In this study, all descriptive data are presented as means ± standard deviations. Independent samples t-test and Mann-Whitney U-test were used to compare differences in basic characteristics between women and men. A mixed-design two-way ANOVA was used to explore the differences in CPR quality between women and men at various phases in WV-CPR and V-CPR. One-Way Repeated Measurement ANOVA was used to analyze differences in CPR quality across all participants in WV-CPR and V-CPR cycle stages. All statistical analyses in this study were performed with IBM SPSS statistical software (version 22, IBM Corp., New York, NY, USA). The statistical significance level was set as *p* less than 0.05.

## Results

### The basic characteristics of subjects

The basic characteristics of all subjects are listed in Table 1. The results of this study showed that women and men in body mass, BMI, PBF, skeletal muscle mass, and handgrip strength were significantly different (all *p* < 0.01).

### CPR quality of V-CPR and WV-CPR tests

The quality of chest compression performed by women and men in WV-CPR and V-CPR is listed in Table 2. Mixed design two-way ANOVA results showed that for the WV-CPR test, both women and men, their CCF and CCD showed significant decreases, and RPE increased significantly with increasing circulatory thoracic pressure time (all *p* < 0.05). For the V-CPR test, women's CCF, CCD, CCRR, and RPE were all significantly changed with the increase in the time of circulating chest compressions (all *p* < 0.05). In men, CCD, CCR, and RPE also had significant changes with increases in the time of cyclic chest compressions (all *p* < 0.05).

### Comparison of CPR quality between V-CPR and WV-CPR tests

**Figure 2:**
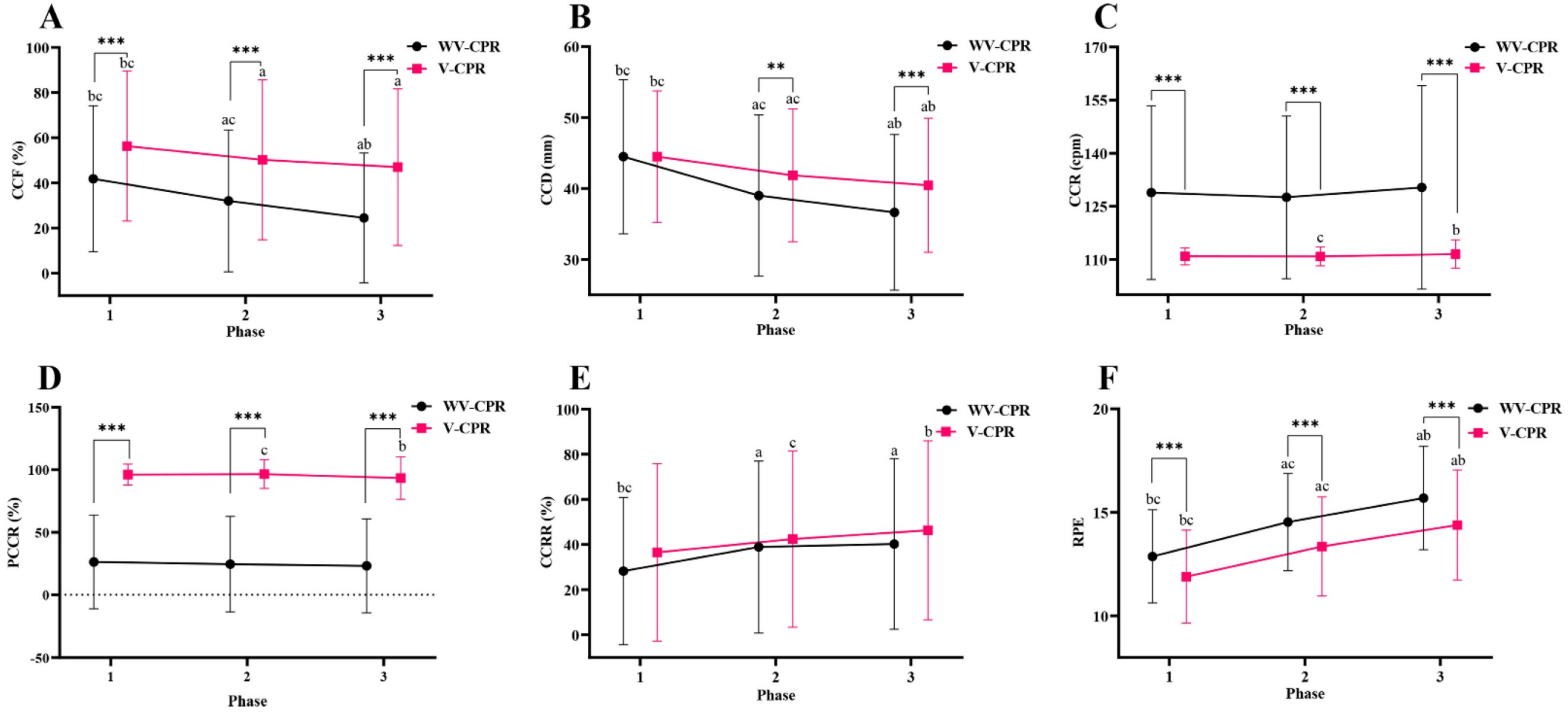
Differences in CCF (**A**), CCD (**B**), CCR (**C**), PCCR (**D**), CCRR (**E**), and RPE (**F**) between WV-CPR and V-CPR (N = 100). WV-CPR, without video-cardiopulmonary resuscitation. V-CPR, video-cardiopulmonary resuscitation. CCF, chest compression fraction. CCD, chest compression depth. CCR, chest compression rate. PCCR, percentage of correct compression rate. CCRR, chest compression rebound rate. RPE, rating of perceived exertion. */**/*** Indicates V-CPR was significantly different from WV-CPR, which represents *p* < 0.05, < 0.01, and < 0.001, respectively. ^a^ Significantly different from phase 1, *p* < 0.05.^ b^ Significantly different from phase 2, *p* < 0.05.^ c^ Significantly different from phase 3, *p* < 0.05.

Differences in CCF, CCD, CCR, PCCR, CCRR and RPE between the WV-CPR and V-CPR in each phase for all subjects (N = 100) were presented in Figure 2. The results of this study showed that CCF, CCR, PCCR, and RPE of phase 1 all achieved significant differences between the WV-CPR and V-CPR (all *p* < 0.001). There were also significant differences between the WV-CPR and V-CPR in CCF, CCD, CCR, PCCR, and RPE of phase 2 and phase 3 (all *p* < 0.01).

### The percentage changes in CPR quality

**Figure 3:**
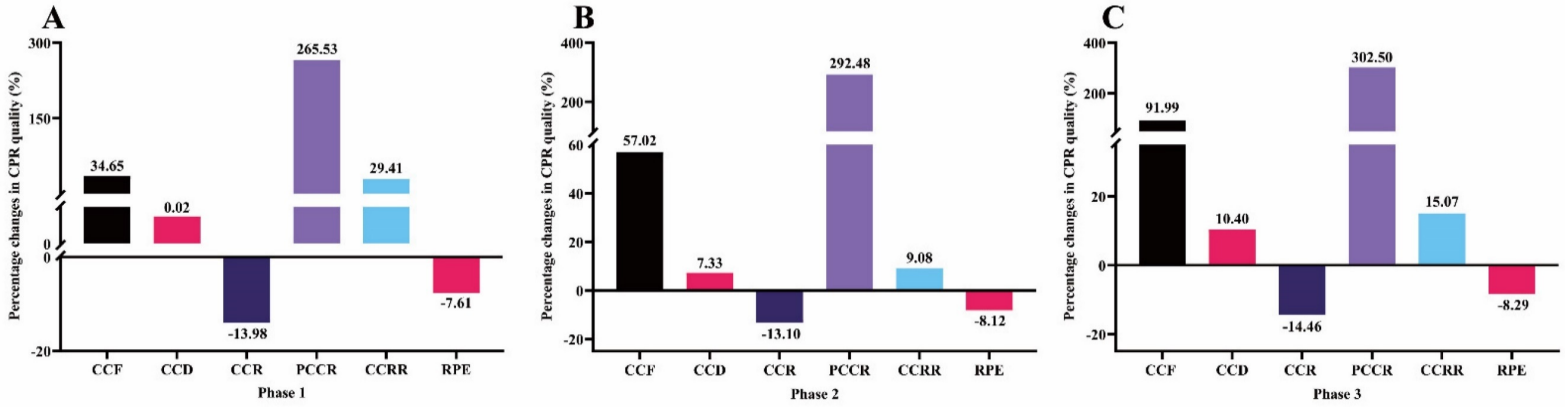
Percentage changes in CPR quality after video intervention (N = 100): (**A**) phase 1; (**B**) phase 2; (**C**) phase 3. CCF, chest compression fraction. CCD, chest compression depth. CCR, chest compression rate. PCCR, percentage of correct compression rate. CCRR, chest compression rebound rate. RPE, rating of perceived exertion.

The percentage changes in CPR quality after video intervention in each phase (N = 100) were presented in Figure 3. The results of this study showed that after video intervention, the subjects' CCF, CCD, PCCR, and CCRR in phase 1 increased by 34.65%, 0.02%, 265.53% and 29.41%, respectively, while CCR and RPE decreased by 13.98% and 7.61%, respectively. The CCF, CCD, PCCR, and CCRR of phase 2 increased by 57.02%, 7.33%, 292.48% and 9.08%, respectively, while the CCR and RPE decreased by 13.10% and 8.12%, respectively. The CCF, CCD, PCCR, and CCRR of phase 3 increased by 91.99%, 10.40%, 302.50% and 15.07%, respectively, while CCR and RPE decreased by 14.46% and 8.29%, respectively.

## Discussion

The primary objective of this study was to assess whether the brief instructional video intervention could improve the quality of CPR performed by inexperienced adults. An additional objective was to compare the differences in CPR performances between men and women. The results of this study confirmed that women and men have significant differences in basic characteristics including PBF, skeletal muscle mass, and handgrip strength, as well as CCF and CCD at various stages of WV-CPR and V-CPR. However, both for women and men, their CCF and CCD in WV-CPR and V-CPR decreased, and RPE scores increased with the increase of chest compression time, which is similar to the findings of Chang et al. 38. However, V-CPR achieved better performance in terms of CCF, CCD, CCR, PCCR and RPE when they were compared with WV-CPR on the whole. Many previous studies have shown that high-quality chest compression is a key factor affecting the survival rate of patients with OHCA 2, 38-40. Therefore, in order to improve the quality of CPR as much as possible, it is recommended that chest compression providers with no experience in CPR practice rescue OHCA patients following the CPR performance steps and rhythm prompts in the video.

CCR is an indicator to measure the quality of chest compressions 41. In clinical studies, CCR was related to the amount of carbon dioxide exhaled 19. Previous studies have shown that providing appropriate CCR during CPR can help improve the return of spontaneous circulation in patients with OHCA 23. According to the latest AHA guidelines, rescuers should maintain a CCR between 100 - 120 compressions per minute (cpm) for 2 min 10, which means no more than 2 compressions per second. However, many previous studies have found that when chest compression is performed without metronome guidance, lower CCR phenomenon is not common, but higher CCR than recommended phenomenon often occurs 19, 22, 27, 28, this study confirmed this phenomenon. In this study, in each 2-min phase, participants performed CCR in WV-CPR (128-130 cpm) exceeding the AHA-specified upper limit of 120 cpm (Figure 2). Many researchers have suggested that fast chest compression leads to more physical fatigue, which ultimately reduces the quality of CPR 19, 27, 41. High CCR may be caused by participants' inability to accurately assess and control CCR. Therefore, in this study, after using 30s video to remind CPR performance points, and then using metronome sound to correct CCR, CCR was within the guideline recommendation (110 cpm), and there was a significant difference in CCR between WV-CPR and V-CPR. In each period, participants performed lower CCR with V-CPR than WV-CPR (Figure 2). Compared with WV-CPR, the PCCR of V-CPR was improved by about 3 times (Figure 3). This shows that it is feasible to assist inexperienced persons to achieve and maintain the CCR recommended by the AHA guidelines by using the reminder of the performance points and the correction of the rhythm sound in the video, thereby improving the CCR accuracy and CPR quality.

CCD affects the relative increase in intrathoracic pressure, which in turn affects positive blood flow from the heart and great vessels to the systemic circulation 2. Therefore, CCD is an important predictor of improved survival and favorable functional outcome after OHCA 44. In the 2020 AHA guidelines, high-quality CPR requires compression depths of at least 50 mm or more, but not more than 60 mm 14. In reality, getting the CCD to this depth is not easy for most chest compression performers 7, 17. A previous study found that during CPR, more than half of the performers had a CCD smaller than 38-51 mm, and 91.6% of the performers had a CCD smaller than 50 mm 21, and the CCD decreased with compression time 26, 45 which are similar to the results of this study. In the present study, the mean CCD of WV-CPR and V-CPR were 44.48 mm and 44.49 mm in the first 2-min phase, respectively, of which 62% and 61% participants had a CCD smaller than 50 mm, respectively. After the first 2-min phase, the CCD of both WV-CPR and V-CPR decreased significantly (Figure 2), but the CCD decrease of WV-CPR was more obvious than that of V-CPR. Moreover, the mean CCD of V-CPR is higher than that of WV-CPR (41.86 vs. 39.00 mm, *p* = 0.002 in the 2^nd^ phase; 40.44 vs. 36.63 mm, *p* < 0.001 in the 3^rd^ phase). Furthermore, consistent with previous findings 17, 18, 26, 27, the average CCD of women was too shallow (WV-CPR: 38.14 mm; V-CPR: 38.80 mm) in the first 2-min phase and significantly lower than that of men's CCD (WV-CPR: 50.82 mm; V-CPR: 50.18 mm). The fact that men have larger body mass (71.46 vs. 57.57 kg), skeletal muscle mass (33.21 vs. 22.46 kg), and handgrip strength (43.38 vs. 26.58 kg) than women may be the reason of this gender difference (all *p* < 0.001; Table 1). Past studies have shown that body weight is one of the predictors of CCD, and men with greater muscle strength and handgrip strength are generally able to generate larger CCDs than women during chest compression 17, 18, 25, while women with lower body weight have difficulty achieving sufficient compression depth 27. These data above suggest that the AHA-recommended CCD (50-60 mm) is somewhat challenging for most non-CPR-experienced individuals, and that the CCD decreases along with the compression time. In the process of CPR execution, the reminder of performance points with video and the correction of rhythm sound can reduce the decline of CCD over time, especially in the 2^nd^ and 3^rd^ phase. Our findings are consistent with the guideline recommendation by the AHA suggesting that men chest compression performers rotate every 2 minutes during CPR. However, women performers need to be replaced more frequently.

CCF is a measure of the proportion of time that compressions are performed throughout resuscitation 2, 10. Frequent and prolonged interruptions in compressions can adversely affect cardiac arrest outcomes 42. Previous studies have shown that a 10% increase in CCF is equivalent to an 11% increase in survival 43. To limit interruptions in compressions during resuscitation, and to maximize blood flow and coronary perfusion, AHA guidelines recommend targeting a CCF of at least 60% 15. In this study, the CCF of V-CPR in the 1^st^, 2^nd^, and 3^rd^ phase was improved by 34.65%, 57.02%, and 91.99% compared with WV-CPR, respectively (Figure 3). Consistent with previous findings 10, we found that CCF decreased with longer resuscitation time (Figure 2A). However, men's mean CCF in V-CPR (72.90% in the 1^st^ phase, 70.14% in the 2^nd^ phase, and 67.34% in the 3^rd^ phase) was within the AHA-recommended CCF range (≥60%) in each 2-min period and significantly higher than WV-CPR (Table 2). These results suggest that reminders of interventional video procedural points and correction of rhythmic tones during CPR contribute to increased CCF in chest compression performers, which in turn improves survival rate in OHCA patients.

Another concern with CPR is rescuers' fatigue. Previous studies have shown that high-quality chest compression is equivalent to a moderate-intensity exercise workload 29. Therefore, continuous chest compressions during CPR can produce fatigue in rescuers, which eventually leads to a gradual decline in CPR quality 7, 11, 24, 32, the results of this study confirmed these points of view. In this study, participants achieved mean RPE in the first 2-min phase of WV-CPR and V-CPR of 13 and 12, respectively, which means CPR is equivalent to the moderate-intensity exercise 46. After 2 minutes, participants' RPE gradually increased (Figure 2F), and CCF (Figure 2A) and CCD (Figure 2B) gradually decreased. Furthermore, the results of this study found that higher compression rates in WV-CPR resulted in more fatigue in participants and increased rates of CCF and CCD descent comparing with the V-CPR (Figure 2), which is consistent with past studies 22, 19. Moreover, compared with WV-CPR in the 2^nd^ and 3^rd^ phase, the RPE of V-CPR decreased by 8.12% and 8.29%, respectively, the CCF increased by 57.02% and 91.99%, and the CCD increased by 7.33% and 10.40%, respectively (Figure 3). To sum up, the reminder of performance points with video during CPR and the correction of rhythm sound can effectively relieve the fatigue of chest compression performers, thereby improving the quality of CPR.

## Limitations

There are some limitations in this study. First, since all participants were adults with no CPR experience and their age was 20.28 ± 2.28 years (rang: 18-30 years), the results in this study cannot be generalized to experienced healthcare providers such as firefighters/emergency medical technicians. Second, the WV-CPR and V-CPR quality tests were conducted in a simulated environment. The Single-rescuer scenario represents one of several OHCA scenarios in which there are no professional healthcare providers on site and only one lay bystander, so CPR can only be performed by one inexperienced adult. The performance attitude of chest compression providers may differ between simulated and actual OHCA situations. Under actual clinical conditions, chest compression providers with higher pressures than in experimental settings may achieve better CPR quality. In addition, both CPR quality tests were performed on manikin, and there are certain differences between manikin and human body, and the quality of chest compression performed on real patients may be very different. Therefore, the findings of this study may not be directly applicable to clinical practice. In the future, more clinical studies are needed to verify these results.

## Conclusions

The primary objective of this study is to examine the role of a brief instructional chest compression-only CPR video in the quality of chest compression for inexperienced adults. The results demonstrated that prolonged continuous chest compressions could lead to fatigue and decreased CPR quality. However, the video intervention could improve chest compression quality by reducing fatigue and CCR, and increasing CCF, CCD and PCCR. To alleviate the fatigue of inexperienced adults and improve their chest compression quality, it is effective and necessary to intervene video performance points and compression rhythm reminders during CPR. With the convenience of modern technology, it can provide efficient and immediate assistance in emergencies. Further clinical trials are required to confirm these results.

## Author Contributions

F.L., C.-S.H., and C.-W.H. designed the study. F.L., C.-W.H., C.-A.H., C.-H.C., C.-Y.W., H.-C.Y., and P.-J.C. carried out the experiments. F.L., C.-S.H., and C.-P.Y. analyzed the data. F.L. and C.-S.H. prepared the figure and tables and wrote the manuscript. All authors have read and agreed to the published version of the manuscript.

## Figures and Tables

**Table 1:** Basic characteristics of subjects

	Total (N = 100)	Men (N = 50)	Women (N = 50)	*P*
Age (years)	20.28 ± 2.28	20.02 ± 1.68	20.54 ± 2.74	0.840^‡^
Height (cm)	168.05 ± 8.38	174.21 ± 5.32	161.88 ± 6.00	< 0.001^†^
Body mass (kg)	64.52 ± 11.38	71.46 ± 9.84	57.57 ± 8.16	< 0.001^†^
BMI (kg/m^2^)	22.74 ± 2.99	23.54 ± 3.00	21.95 ± 2.79	0.007^†^
PBF (%)	22.59 ± 7.76	17.49 ± 5.56	27.69 ± 6.12	< 0.001^†^
Skeletal muscle mass (kg)	27.84 ± 6.29	33.21 ± 3.78	22.46 ± 2.58	< 0.001^‡^
Handgrip strength (kg)	34.98 ± 10.42	43.38 ± 6.78	26.58 ± 5.43	< 0.001^†^

Values are presented as the mean ± SD. BMI, body mass index. PBF, percentage of body fat. ^†^ *p*-value for independent samples t-test between men and women; ^‡^ *p*-value for the Mann-Whitney U test between men and women.

**Table 2:** Comparison of the quality of chest compression between WV-CPR and V-CPR

	Women (N = 50)			Men (N = 50)		
	WV-CPR	V-CPR	*p*	WV-CPR	V-CPR	*p*
**Phase 1**						
CCF (%)	30.14 ± 30.35^bc,†^	39.72 ± 30.58^bc,‡^	0.015	53.50 ± 30.13^bc^	72.90 ± 27.06	< 0.001
CCD (mm)	38.14 ± 10.62^bc,†^	38.80 ± 8.18^bc,‡^	0.494	50.82 ± 6.59^bc^	50.18 ± 6.38^bc^	0.507
CCR (cpm)	130.95 ± 22.21	110.58 ± 2.05	< 0.001	126.77 ± 26.70	111.12 ± 2.69^c^	< 0.001
PCCR (%)	24.08 ± 35.24	96.52 ± 4.94^c^	< 0.001	28.54 ± 39.97	95.82 ± 10.93	< 0.001
CCRR (%)	38.74 ± 35.45^bc,†^	47.84 ± 40.46^c‡^	0.089	17.64 ± 25.84^bc^	25.12 ± 35.00	0.162
RPE	13.12 ± 2.23^bc^	12.22 ± 2.08^bc^	0.001	12.62 ± 2.28^bc^	11.56 ± 2.37^bc^	< 0.001
**Phase 2**						
CCF (%)	19.02 ± 27.46^ac,†^	30.32 ± 30.79^a,‡^	0.002	44.96 ± 29.93^ac^	70.14 ± 27.82	< 0.001
CCD (mm)	32.16 ± 10.62^ac,†^	36.04 ± 7.62^ac,‡^	< 0.001	45.84 ± 7.25^ac^	47.68 ± 7.05^ac^	0.075
CCR (cpm)	127.87 ± 20.76	110.54 ±1.98	< 0.001	127.22 ± 25.24	111.14 ± 3.19^c^	< 0.001
PCCR (%)	23.20 ± 37.68	97.66 ± 5.62^c^	< 0.001	26.02 ± 39.01	95.52 ± 15.29	< 0.001
CCRR (%)	49.14 ± 39.38^a,†^	55.38 ± 38.92^‡^	0.201	28.60 ± 34.17^ac^	29.42 ± 35.09	0.866
RPE	14.56 ± 2.21^ac^	13.40 ± 2.36^ac^	< 0.001	14.50 ± 2.50^ac^	13.30 ± 2.45^ac^	< 0.001
**Phase 3**						
CCF (%)	14.70 ± 25.77^ab,†^	26.58 ± 28.50^a,‡^	0.001	34.22 ± 28.50^ab^	67.34 ± 27.85	< 0.001
CCD (mm)	30.60 ± 10.22^ab,†^	34.38 ± 7.67^ab,‡^	< 0.001	42.66 ± 8.02^ab^	46.50 ± 6.80^ab^	< 0.001
CCR (cpm)	131.10 ± 25.03	110.72 ± 2.65	< 0.001	129.59 ± 32.24	112.28 ± 4.95^ab^	< 0.001
PCCR (%)	20.40 ± 34.06	95.50 ± 11.10^ab^	< 0.001	26.02 ± 40.87	91.34 ± 21.23	< 0.001
CCRR (%)	46.82 ± 39.73^a^	58.90 ± 38.59^a,‡^	0.014	33.62 ± 34.95^ab^	33.66 ± 37.03	0.993
RPE	15.74 ± 2.29^ab^	14.26 ± 2.64^ab^	< 0.001	15.64 ± 2.72^ab^	14.52 ± 2.70^ab^	< 0.001

Values are shown as the mean ± SD. WV-CPR, without video-cardiopulmonary resuscitation. V-CPR, video-cardiopulmonary resuscitation. CCF, chest compression fraction. CCD, chest compression depth. CCR, chest compression rate. PCCR, percentage of correct compression rate. CCRR, chest compression rebound rate. RPE, rating of perceived exertion. ^a^ Significantly different from phase 1, *p* < 0.05.^ b^ Significantly different from phase 2, *p* < 0.05.^ c^ Significantly different from phase 3, *p* < 0.05. ^†^ Indicates women were significantly different from men in WV-CPR, *p* < 0.05.^ ‡^ Indicates women were significantly different from men in V-CPR, *p* < 0.05.

## Abbreviations

AHA: American Heart Association
BMI: body mass index
CPR: cardiopulmonary resuscitation
CCF: chest compression fraction
CCR: chest compression rate
CCD: chest compression depth
CCRR: chest compression rebound rate
DA-CPR: dispatch-assisted CPR
EMS: emergency medical services
OHCA: out-of-hospital cardiac arrest
PBF: percentage of body fat
PCCR: percent of correct compression rate
RPE: rating of perceived exertion
V-CPR: video-CPR
WV-CPR: without video-CPR
